# Trehalose promotes the survival of random-pattern skin flaps by TFEB mediated autophagy enhancement

**DOI:** 10.1038/s41419-019-1704-0

**Published:** 2019-09-15

**Authors:** Hongqiang Wu, Huanwen Chen, Zhilong Zheng, Jiafeng Li, Jian Ding, Zihuai Huang, Chang Jia, Zitong Shen, Guodong Bao, Lingyun Wu, Abdullah Al Mamun, Huazi Xu, Weiyang Gao, Kailiang Zhou

**Affiliations:** 1 Department of Orthopaedics, The Second Affiliated Hospital and Yuying Children’s Hospital of Wenzhou Medical University, Wenzhou, 325027 China; 2 Zhejiang Provincial Key Laboratory of Orthopaedics, Wenzhou, 325027 China; 3 The Second Clinical Medical College of Wenzhou Medical University, Wenzhou, 325027 China; 4 University of Maryland School of Medicine, Baltimore, MD 21201 USA; 5 School of Pharmaceutical Science, Wenzhou Medical University, Wenzhou, 325027 China; 6 Pediatric Research Institute, The Second Affiliated Hospital and Yuying Children’s Hospital of Wenzhou Medical University, Wenzhou, 325027 China; 7 Renji College of Wenzhou Medical University, Wenzhou, 325027 China; 8 Department of Respiration, The Second Affiliated Hospital of Jiaxing University, Jiaxing, 314001 China

**Keywords:** Pharmaceutics, Trauma

## Abstract

Random-pattern skin flaps are commonly used and valuable tools in reconstructive surgery, however, post-operative random skin flap necrosis remains a major and common complication. Previous studies have suggested that activating autophagy, a major pathway for degradation of intracellular waste, may improve flap survival. In this study, we investigated whether trehalose, a novel and potent autophagy activator, improves random skin flap viability. Our results demonstrated that trehalose significantly improves viability, augments blood flow, and decreases tissue edema. Furthermore, we found that trehalose leads to increased angiogenesis, decreased apoptosis, and reduced oxidative stress. Using immunohistochestry and western blot, we demonstrated that trehalose augments autophagy, and that inhibition of autophagy augmentation using 3MA significantly blunted the aforementioned benefits of trehalose therapy. Mechanistically, we showed that trehalose’s autophagy augmentation is mediated by activation and nuclear translocation of TFEB, which may be due to inhibition of Akt and activation of the AMPK-SKP2-CARM1 signaling pathway. Altogether, our results established that trehalose is a potent agent capable for significantly increasing random-pattern skin flap survival by augmenting autophagy and subsequently promoting angiogenesis, reducing oxidative stress, and inhibiting cell death.

## Introduction

Random-pattern skin flaps are commonly used in reconstructive surgery to repair skin defects due various causes such as trauma, congenital disorders, cancer, and diabetes mellitus^1–3^. However, random-pattern flaps have a non-specific (or “random”) blood supply, making postoperative flap necrosis a frequent complication. The lack of specific arteriovenous system and blood supply is particularly problematic for distal regions of flaps^4,5^, and flap survival relies on angiogenesis starting from flap pedicle towards distal areas. Inadequate blood supply and subsequent ischemia-reperfusion-injury can lead to significant oxidative stress and apoptosis^6–8^, ultimately resulting in flap death. Given the common use of random-pattern skin flaps and the high frequency of flap necrosis, strategies to promote angiogenesis, alleviate oxidative stress, and reduce cell death have been under active investigation in recent years^7,9,10^.

Autophagy is a process by which intracellular contents are degraded by the cell’s own lysosomal system in autophagic vesicles^11^. Previous studies have shown that autophagy is a critical mechanism that can promote angiogenesis^12,13^, alleviate oxidative stress, and inhibit apoptosis^14^. Our studies in a rat skin flap model confirmed that autophagy can act through these mechanisms to promote flap survival, highlighting the potential for using autophagy activating agents to improve outcomes^8^.

Trehalose (TRE), a natural, non-reducing a-linked disaccharide (a, a-1,1-glucoside), has been identified as a potent mTOR-independent autophagy enhancer, and plays an essential role in cell survival and maintenance through activating autophagy^15^. Previous studies have found various therapeutic uses of trehalose, such as in atherosclerosis where TRE-induced autophagy enhances the function of macrophage autophagy-lysosomal system to reduce atherosclerotic plaque burden^16^. In a cell model of Amyotrophic Lateral Sclerosis, TRE induces neuronal autophagy and accelerates the removal of TAR DNA-binding protein-43^17^. TRE can also reduce cardiac hypertrophy, apoptosis, and fibrosis in chronic ischemic remodeling via activating autophagy^18^. Despite its obvious promise, trehalose’s effect on autophagy is a recent discovery, and it remains a largely under-investigated agent. Whether trehalose can exert beneficial effects on tissue survival after reconstructive grafting is completely unknown. Thus, the present study seeks to investigate whether trehalose can promote random pattern skin flap survival via autophagy augmentation and to explore its mechanism of action.

## Materials and methods

### Experimental animals

One hundred and ninety-two healthy C57BL/6 mice (male, average weight 20–30 g) were purchased from Wenzhou Medical University’s Experimental Animal Center (License no. SCXK 2005–0019), Zhejiang Province, China. Temperature of 22–25 °C, humidity of 60–70%, and 12 h light: 12 h dark cycles were applied as standard environmental conditions where animals were housed, and mice were given free access to food and water. The animals used in this study were approved by Wenzhou Medical University’s Animal Research Committee (wydw2017-0022) and cared in accordance with the ethical guidelines on animal experimentation of Laboratory Animals of China National Institutes of Health. To perform our study, Animals were randomly divided into six groups: Control (*n* = 36), sucrose (SUC, *n* = 36), TRE (*n* = 36), 3-methyladenine (3MA, *n* = 18), TRE+3MA (*n* = 18), TRE + adeno-associated virus (AAV)- Scramble control (TRE+ Scramble control, *n* = 24), and TRE+AAV−TFEB short hairpin RNA (TRE+TFEB shRNA, *n* = 24).

### Reagents and antibodies

The following reagents and antibodies and their suppliers were acquired as follows: Solarbio Science & Technology (Beijing, China): Trehalose (C_12_H_22_O_11_·2H_2_O; purity ≥ 99.5%), Sucrose (C_12_H_22_O_11_; purity > 99.9%), H&E Staining Kit, DAB developer, and pentobarbital sodium. Boster Biological Technology (Wuhan, China): Cadherin 5 primary antibody. Biogot Technology (Shanghai, China): GAPDH primary antibody. Protein tech Group (Chicago, IL, USA): VEGF, Superoxide Dismutase 1 (SOD1), Vacuolar Protein Sorting 34 (VPS34), Matrix Metalloproteinase 9 (MMP9), Heme Oxygenase 1 (HO1), Cathepsin D (CTSD), Caspase 3 (CAPS3), Histone-H3, Akt and SKP2 primary antibodies. Cell Signaling Technology (Beverly, MA, USA): Cytochrome C (CYC), Bax, AMPK, p-AMPK, Endothelial Nitric Oxide Synthase (eNOS), FOXO3a, p-FOXO3a, and CARM1 primary antibodies. Abcam (Cambridge, UK): SQSTM1/p62 and CD34 primary antibodies. Sigma-Aldrich Chemical Company (Milwaukee, WI, USA): Microtubule-associated proteins 1A/1B light chain 3 (LC3B) primary antibody and 3MA (C_6_H_7_N_5_; purity ≥ 98.00%,). Bethyl Laboratories (Montgomery, TX, USA): TFEB primary antibody. Signalway Antibody (College Park, MD, USA): p-Akt primary antibody. Boyun Biotechnology (Nanjing, China): Fluorescein isothiocyanate (FITC)-conjugated IgG secondary antibody. Beyotime Biotechnology (Jiangsu, China): 4′,6-Diamidino-2-phenylindole (DAPI) solution. Thermo Fisher Scientific (Rockford, IL, USA): The BCA Kit, NE-PER Nuclear and Cytoplasmic Extraction Reagents, and Pierce Co-Immunoprecipitation Kit. PerkinElmer Life Sciences (Waltham, MA, USA): The ECL Plus Reagent Kit.

### Flap animal model

Intraperitoneal injection of 1% (w/v) pentobarbital sodium was used to anesthetize each animal (50 mg/kg). Then, electric shaver and depilatory cream was used to remove dorsal fur. A 1.5 by 4.5 cm caudally- based random-pattern flap was elevated in the mouse dorsum beneath the pannculus carnosus as previously described^19^. All known vessels were completely transected, and 4-0 non-absorbable sutures were immediately put in place to secure the flaps in their original position. Three separate equal zones were demarcated on each flap: Area I (most proximal with respect to the caudal base of the flap), Area II, and Area III (most distal).

### AAV vector packaging

Shanghai Genechem Company (Shanghai, China) constructed and packaged the AAV-TFEB shRNA used in our experiments. The shRNA sequence of protein kinase, TFEB-activated was synthesized and cloned into pAV-U6-shRNA-CMV-EGFP plasmid to produce pAV-U6-shRNA (TFEB)-CMV-EGFP. AAV9-U6-shRNA (TFEB)-CMV-EGFP was produced by transfection of AAV-293 cells with pAV-U6-shRNA (TFEB)-CMV-EGFP, adenovirus helper plasmid (Ad helper), and AAV Rep/Cap expression plasmid. With similar process, AAV9-U6-shRNA (scramble)-CMV-EGFP was produced as Scramble control. Viral particles were purified by iodixanol gradient method. The titer of AAV9-U6-shRNA (TFEB)-CMV-EGFP and AAV9-U6-shRNA (scramble)-CMV-EGFP was 1.243 × 10^12^, 1.22 × 10^12^ genomic copies per ml, respectively, determined by quantitative PCR.

### Drugs and AAV vectors administration

Daily intraperitoneal injection of 2 g/kg trehalose was administered starting 12 days before operation for the TRE group, and continued until the animals were euthanized. While both intraperitoneal (i.p. 2 g/kg, daily) and oral (3% w/v, ad libitum) trehalose administration have been commonly used in in prior studies of various diseases in animal models^20–23^, recent research reports that the i.p. administration of TRE leads to far greater elevations inserum trehalose than the oral dosing^20^. Furthermore, i.p. administration can easily equate to intravenous administration, which would be very convenient for patients and hospital staff in the pre- and post-op patient, especially if patients need to be NPO (nothing by mouth) for any reason (e.g. clinical status, peri-operative considerations, etc.). Thus, we performed our studies using i.p. administration of trehalose. 2 g/kg sucrose and saline were administered in an identical fashion for the SUC group and control group, respectively. Daily intraperitoneal injection of 15 mg/kg 3MA was performed in an identical fashion for the 3MA group. Daily intraperitoneal injection of 15 mg/kg 3MA were administered 30 min before trehalose administration for the TRE + 3MA group. The TRE+Scramble control group and the TRE + TFEB shRNA group received 6 daily micro ml subcutaneous injections of viral vectors in PBS with 5 × 10^9^ packaged genomic particles total for 3 areas 14 days before operation (2 days before trehalose administration); the TRE+TFEB ShRNA and TRE+Scramble groups then continued with trehalose injections with the same protocol as those for the TRE group.

### Flap survival evaluation

Flat viability was evaluated via high-quality photography on postoperative day (POD) 3 and 7. Viable and ischemic areas were identified using Image-Pro Plus imaging software (ver. 6.0; Media Cybemetics), and % viable area was calculated as: x 100%.

### Laser doppler blood flow (LDBF) imaging

To measure blood flow, 6 mice in each group were placed under anesthesia and scanned using a laser doppler instrument (Moor Instruments, Axminster, UK) on POD 7. LDBF measurement protocols were previously described^24^. Blood flow was quantified using perfusion units, and were calculated using the Moor LDI Review software (ver.6.1; Moor Instruments). Each animal was scanned and measured 3 times, and the average value was used for further statistical analysis.

### Tissue edema measurement

Tissue edema was measured with the following protocol. On POD7, skin flaps from six animals in each group was removed and weighed to obtain the “wet weight.” Then, these flaps were placed in an autoclave at 50 degrees Celsius for dehydration, and were allowed to stabilize for 2 days. Then, flaps were weighed again to obtain the “dry weight.” Edema was reported as the percentage of water content on POD7, and is calculated with the following formula: (/wet weight) × 100%.

### Hematoxylin and eosin (H&E) staining

After animals were euthanized on POD7, six 1 cm by 1 cm tissue specimen were retrieved from Area II of skin flaps (intermediate location). These specimens were then sectioned transversely after fixing with 4% paraformaldehyde and embedding in paraffin wax. H&E staining was then performed on 4 µm thick sections mounted on poly-L-lysine-coated slides. Under x200 light microscopy (Olympus Corp, Tokyo, Japan), microvessels were enumerated on 6 randomly selected fields from three random sections, and number of microvessels per unit area (/mm^2^) was calculated to quantify vessel density.

### Immuno histochemistry (IHC)

Six paraffinized sections described in section regarding H&E staining were deparaffinized using xylene and rehydrated through a graded ethanol bath for immunohistochemical analyses. Sections were washed and then blocked with 3% (v/v) H_2_O_2_. Then, sections were placed in 10.2 mM sodium citrate buffer at 95 degrees Celsius for 20 min, and then blocked with 10% (w/v) bovine serum albumin phosphate buffered saline for 10 min. Finally, sections were incubated overnight at 4 degrees Celsius with the following primary antibodies: anti-CD34 (1:100), anti-VEGF (1:300), anti-Cadherin 5 (1:100), anti-CASP3 (1:200), anti-SOD1 (1:100), and anti-CTSD (1:100). Next, sections were incubated with HRP-conjugated secondary antibody and counterstained with hematoxylin. Using a DP2-TWAN image-acquisition system (Olympus Corp, Tokyo, Japan), flap tissues were imaged at ×200 magnifications, and Image-Pro Plus (Media Cybernetics, Rockville, MD, USA) quantified absorption values to estimate VEGF, Cadherin 5, CASP3, SOD1, and CTSD expressions. The density of CD34-positive blood vessels was calculated using similar methods as described in section 2.9. Overall, six random fields of three random sections were included for IHC analysis.

### Immunofluorescence staining

Similar to methods described in the section regarding IHC, sections were deparaffinized, rehydrated, washed, and then treated with 10.2 mM sodium citrate buffer for 20 min at 95 °C. Then, 0.1% (v/v) PBS-Triton X-100 were used to permeabilize the sections for 30 min. After 1 h of blocking with 10% (v/v) bovine serum albumin in PBS, specimens were incubated with primary antibodies against TFEB (1:100) and LC3II (1:200) overnight at 4 °C overnight. Following this, specimens were incubated at room temperature for 1 h with FITC-conjugated secondary antibody. Fluorescence microscope (Olympus, Tokyo, Japan) was used to visualize and evaluate the dermal layer of 6 randomly selected fields from 3 random sections of each specimen, and the percentage of LC3II-positive cells and TFEB translocation into nucleus was calculated.

### Western blotting and co-immuno precipitation

On POD 7, six 0.5 cm by 0.5 cm skin samples were retrieved from the middle section of Area II and stored at −80 degrees Celsius for Western blotting and co-immunoprecipitation analyses. For coimmunoprecipitation (Co-IP) analysis, samples from the control, SUC, and TRE groups were processed according to the protocol provided by the manufacturer (Pierce). Next, coupling resin was used to immobilize antibodies, and mixed with flap lysate for storage at 4 degrees Celsius overnight. Then, we eluted the proteins for western blotting. In addition, six samples in each group were processed by extracting proteins with a lysis buffer. Another six samples in each group were processed by extracting cytoplasmic protein and nuclear protein with NE-PER (Nuclear and Cytoplasmic Extraction Reagents). Protein concentrations were calculated using the BCA assay. Proteins were separated by 12% polyacrylamide gel electrophoresis and subsequently transferred to PVDF membranes. After blocking with 5% (w/v) nonfat milk, the membranes were incubated overnight with the following primary antibodies at 4 °C overnight: VEGF (1:1000), MMP-9 (1:1000), Cadherin 5 (1:1000), HO-1 (1:1000), eNOS (1:1000), SOD1 (1:1000), Bax (1:1000), CYC (1:1000), CASP3 (1:1000), BECN1/Beclin1 (1:1000), SQSTM1/p62 (1:1000), LC3II (1:1000), VPS34 (1:1000), CTSD (1:1000), AMPK (1:1000), p-AMPK (1:1000), FOXO3a (1:1000), p-FOXO3a (1:1000), SKP2 (1:1000), CARM1 (1:1000), Akt (1:1000), p-Akt (1:1000), TFEB (1:1000), GAPDH (1:1000), and Histone-H3 (1:1000). Then the membranes were incubated with secondary antibodies for 2 h at room temperature. The immunoreactive proteins were visualized using the ECL Plus Reagent Kit. Finally, the band intensity was quantified using Image Laboratory 3.0 software (Bio-Rad Laboratories Inc, Hercules, CA, USA).

### Real-time polymerase chain reaction (PCR)

Six 0.5 cm by 0.5 cm skin samples of Area II were obtained for real-time PCR analysis. Total of RNA was collected from the flaps after treatment with Tizol reagent. Next, cDNA was synthesized by reverse transcriptase according to manufacturer’s instructions of Prime Script II 1st Strand cDNA Synthesis Kit (6210B, TAKARA BIO INC). Primers were designed using Primer Premier 5.0 according the mRNA sequences of BECN1/Beclin1, VPS34, CTSD, SQSTM1/p62 and LC3 checked in GenBank, and synthesized by Nanjing Zoonbio Biotechology Co.,Ltd. Primer sequences are as follows: Beclin1 5′-ATGGAGGGGTCTAAGGCGTC-3′ (forward) and 5′-TGGGCTGTGGTAAGTAATGGA-3′ (reverse); VPS34 5′-TAACGTGGAGGCAGATGGTT-3′ (forward) and 5′-CATGTGTCCTTGCCGATGAG-3′ (reverse); CTSD 5′-GGGCATCCAGGTAGTTTT-3′ (forward), and 5′-CGTCTTGCTGCTCATTCT-3′ (reverse); SQSTM1/p62 5′-ACAACCCGTGTTTCCTTT-3′ (forward), and 5′-TGCCACCTTTCACTCACTA-3′ (reverse); LC3 5′-CTACGCCTCCCAAGAAACC-3′ (forward), and 5′-AGAGCAACCCGAACATGACT-3′ (reverse); TFEB 5′-CAGCAGGTGGTGAAGCAAGAGT*-*3′ (forward) and 5′-TCCAGGTGATGGAACGGAGACT*-*3′ (reverse);β-actin 5′-ATGTGGATCAGCAAGCAGGA-3′ (forward), and 5′-AAGGGTGTAAAACGCAGCTCA-3′(reverse). Real-time PCR was performed using SYBR Premix Ex Taq (RR420A, TAKARA BIO INC). The reaction conditions included denaturation at 95 °C for 30 s, annealing at 65 °C for 30 s, and extension at 72 °C for 45 s for 30 cycles. Finally, the signal was detected at 72 °C. Beclin1, VPS34, CTSD, SQSTM1/p62 and LC3 mRNAs were normalized to β-actin mRNA.

### Statistical analysis

SPSS software version 19.0 (SPSS, Chicago, IL) was used for all statistical analyses. Data are presented as mean ± Standard Error of Mean (SEM). Independent-sample *t*-test and one-way ANOVA with LSD (equal variances assumed) or Dunnett’s T3 (equal variances not assumed) post-hoc analyses were used as appropriate. P-values less than 0.05 is considered statistically significant.

## Results

### TRE improves skin flap survival

**Fig. 1:**
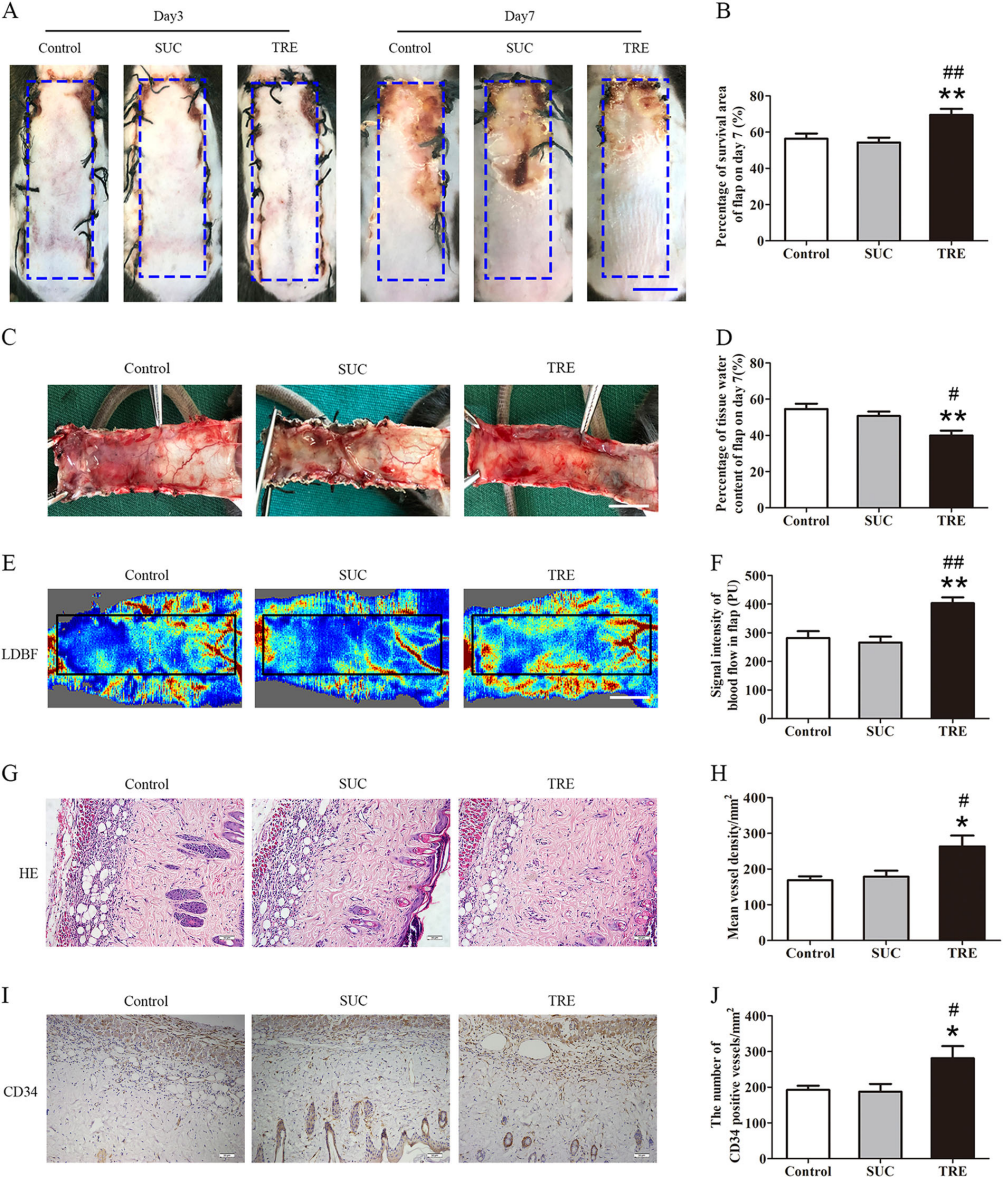
TRE enhances the survival of random skin flaps. **a** Digital photographs of flaps of the Control, SUC and TRE groups on POD 3 and POD 7 (scale bar, 1 cm). **b** The percentages of survival area in the Control, SUC and TRE groups were quantified and analyzed. **c** Digital photographs of the inner side of flaps in the Control, SUC and TRE groups on POD7 (scale bar, 1 cm). **d** Histogram of percentage of tissue water content in each group. **e** Full field LDBF images of flaps in each group on POD 7 (scale bar, 1 cm). **f** The signal intensity of blood flow of flaps was quantified and analyzed. **g** H&E staining to show vessels in area II of flaps in the Control, SUC and TRE groups (original magnifcation×200; scale bar, 50 μm). **h** Histogram of percentage of MVDs in each group. **(i)** IHC for CD34 to present vessels of area II in the Control, SUC and TRE groups (original magnifcation×200; scale bar, 50 μm). **j** Histogram of percentage of CD34-positive vessels in each group. Values are expressed as means ± SEM, *n* = 6 per group. *p < 0.05 and **p < 0.01, vs. Control group; ^#^*p* < 0.05 and ^##^*p* < 0.01, vs. SUC group

The non-reducing nature of sucrose (SUC) makes it the ideal control disaccharide in comparison to trehalose^20^. Therefore, the SUC group was used to establish negative control, and were expected to yield results similar to the Control group. Three days after surgical procedure, skin flaps in all groups showed no obvious necrosis in Area III but did appear edematous and pale. Seven days after surgery, while survival is evident in Area I in all groups, Area III began to display signs of necrosis with darkening, hardening, and scabbing, with some similar findings spreading to Area II flap (Fig. 1a). The TRE group showed significantly superior survival than Control and SUC groups (Fig. 1b). Qualitatively, significant edema and subcutaneous venous congestion were appreciated in Control and SUC groups, but to a lesser extent in the TRE group (Fig. 1c). Quantitatively, the TRE group showed lower water content (Fig. 1d). Blood flow in the TRE group was improved (Fig. 1e), and quantitative blood flow measurements were also significantly superior (Fig. 1f). For angiogenesis, we found significantly more microvessels in the TRE group (Fig. 1g), with higher mean vessel density than the Control and SUC group (Fig. 1h). The TRE group also showed more CD34+ vessels (Fig. 1i, j). Meanwhile, there was no significant difference between the Control and SUC groups in the above tests. Together, these data suggest that trehalose improves the survival of skin flaps.

### TRE promotes angiogenesis in random skin flaps

**Fig. 2:**
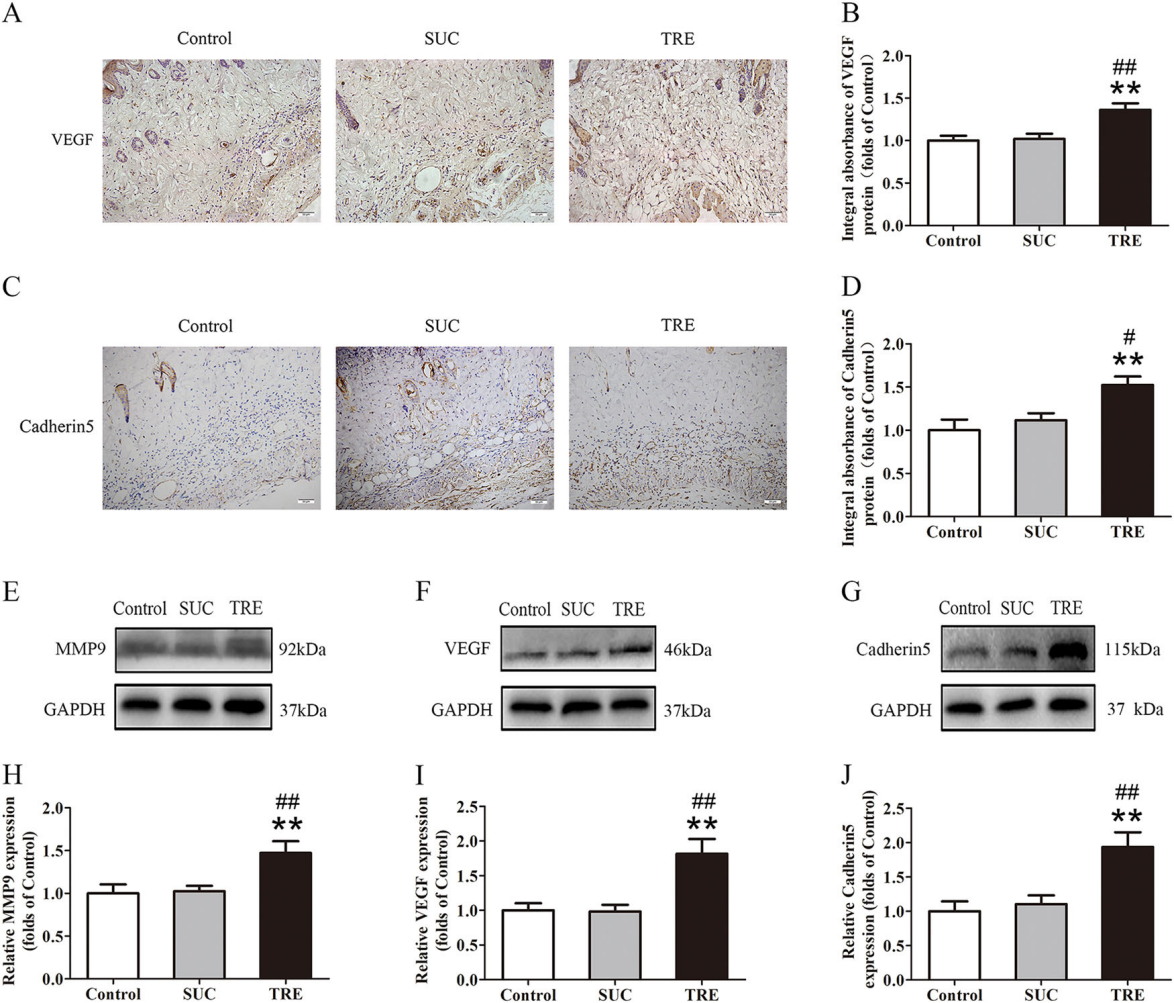
TRE promotes angiogenesis in random skin flaps. **a**, **c** IHC for VEGF and Cadherin 5 expressions in the ischemic flaps of the Control, SUC and TRE groups (original magnification × 200; scale bar, 50 μm). **b**, **d** The optical density values of VEGF and Cadherin 5 were quantified and analyzed in each group. **e**–**g** Western blotting for MMP9, VEGF, and Cadherin 5 expressions in the ischemic flaps of the Control, SUC and TRE groups. The gels have been run under the same experimental conditions, and cropped blots are used here. **h**–**j** Optical density values of MMP9, VEGF, and Cadherin 5 were quantified and analyzed in each group. Values are shown as means ± SEM, *n* = 6 per group. **p < 0.01, vs. Control group; ^#^*p* < 0.05 and ^##^*p* < 0.01, vs. SUC group

Blood supply, and thus angiogenesis, is a critical component that determines skin flap viability, thus, we asked whether trehalose’s positive effects on skin flap survival may be mediated by increased angiogenesis. VEGF and Cadherin 5 expression was evaluated by IHC. In the TRE group, VEGF expression in Area II of skin flaps is significantly higher than in Control and SUC groups (Fig. 2a, b). These findings were corroborated with western blot analysis (Fig. 2f, i). Endothelial and stromal cell Cadherin 5 expression was also increased in the TRE (Fig. 2c, d). This result was confirmed with western blotting showing similar differences (Fig. 2g, j). We used western blotting to further evaluate MMP9 protein levels. Here, we also found that levels were significantly increased in the TRE group compared with Control and SUC groups (Fig. 2e, h). Meanwhile, there was no significant difference between the Control and SUC groups in the above tests. Together, these data suggest that trehalose promotes angiogenesis in skin flaps.

### TRE decreases apoptosis in random skin flaps

**Fig. 3:**
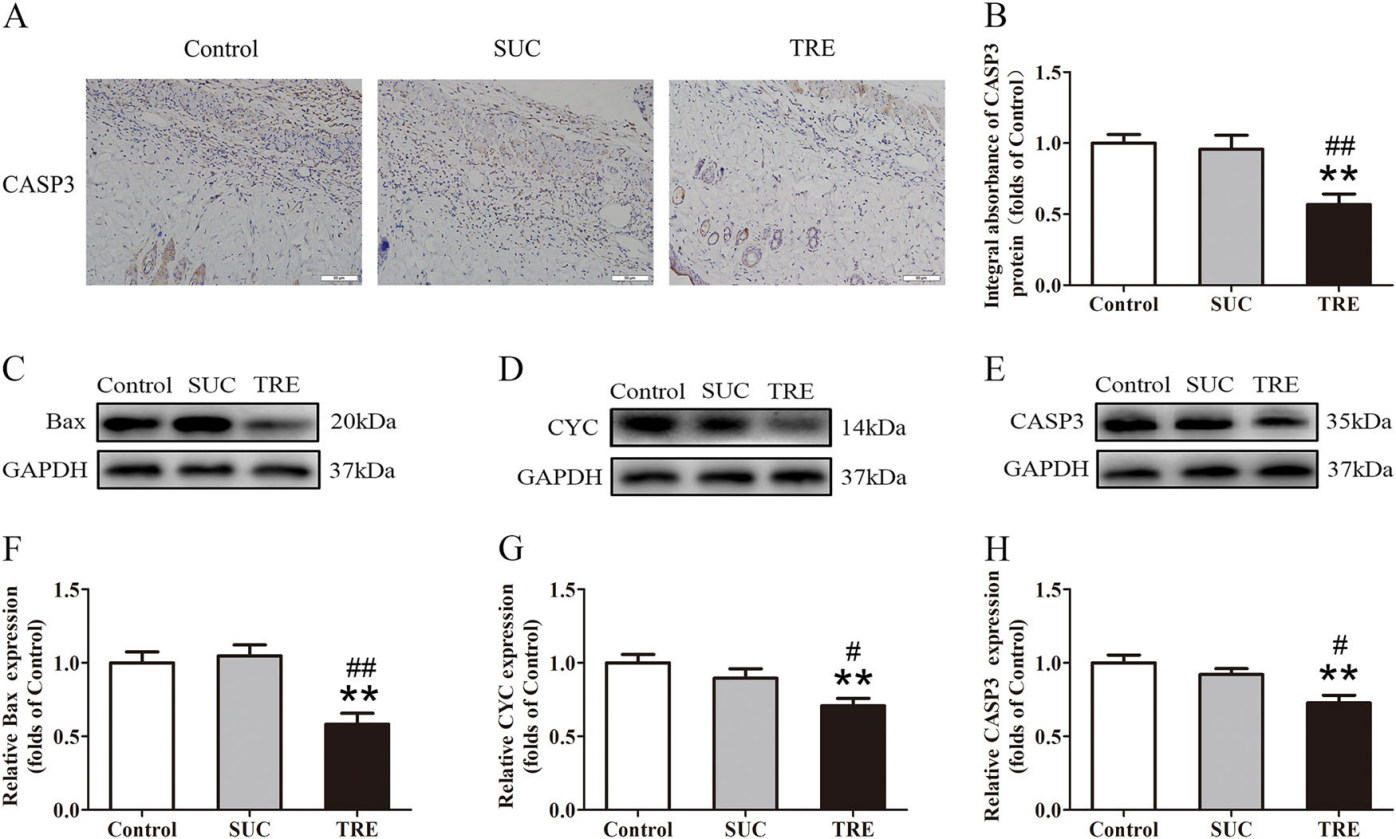
TRE reduces apoptosis in random skin flaps. **a** IHC for CASP3 expression in the ischemic flaps of the Control, SUC and TRE groups (original magnification ×200; scale bar, 50 μm). **b** The optical density values of CASP3 were quantified and analyzed in each group. **c**–**e** Western blotting for Bax, CYC, and CASP3 expressions in the ischemic flaps of the Control, SUC and TRE groups. The gels have been run under the same experimental conditions, and cropped blots are used here. **f**–**h** Optical density values of Bax, CYC and CASP3 were quantified and analyzed in each group. Values are presented as means ± SEM, *n* = 6 per group. *p < 0.05 and **p < 0.01, vs. Control group; ^#^*p* < 0.05 and ^##^*p* < 0.01, vs. SUC group

Next, we investigated whether trehalose modulates apoptosis in skin flaps. CASP3 levels in the dermis as measured by IHC and integral absorbance analyses revealed significant decrease in the TRE group compared with Control and SUC groups (Fig. 3a, b), consistent with that seen in western blotting (Fig. 3e, h). Levels detected by IHC and western blotting were not different between Control and SUC groups (Fig. 3a, b, e, h). Furthermore, western blotting revealed significant decreases in Bax and CYC levels in the TRE group relative to the Control and SUC group, while no differences between the Control and SUC group (Fig. 3c, d, f, g). Altogether, these results demonstrate that trehalose decreases apoptosis in skin flaps.

### TRE reduces oxidative stress in random skin flaps

**Fig. 4:**
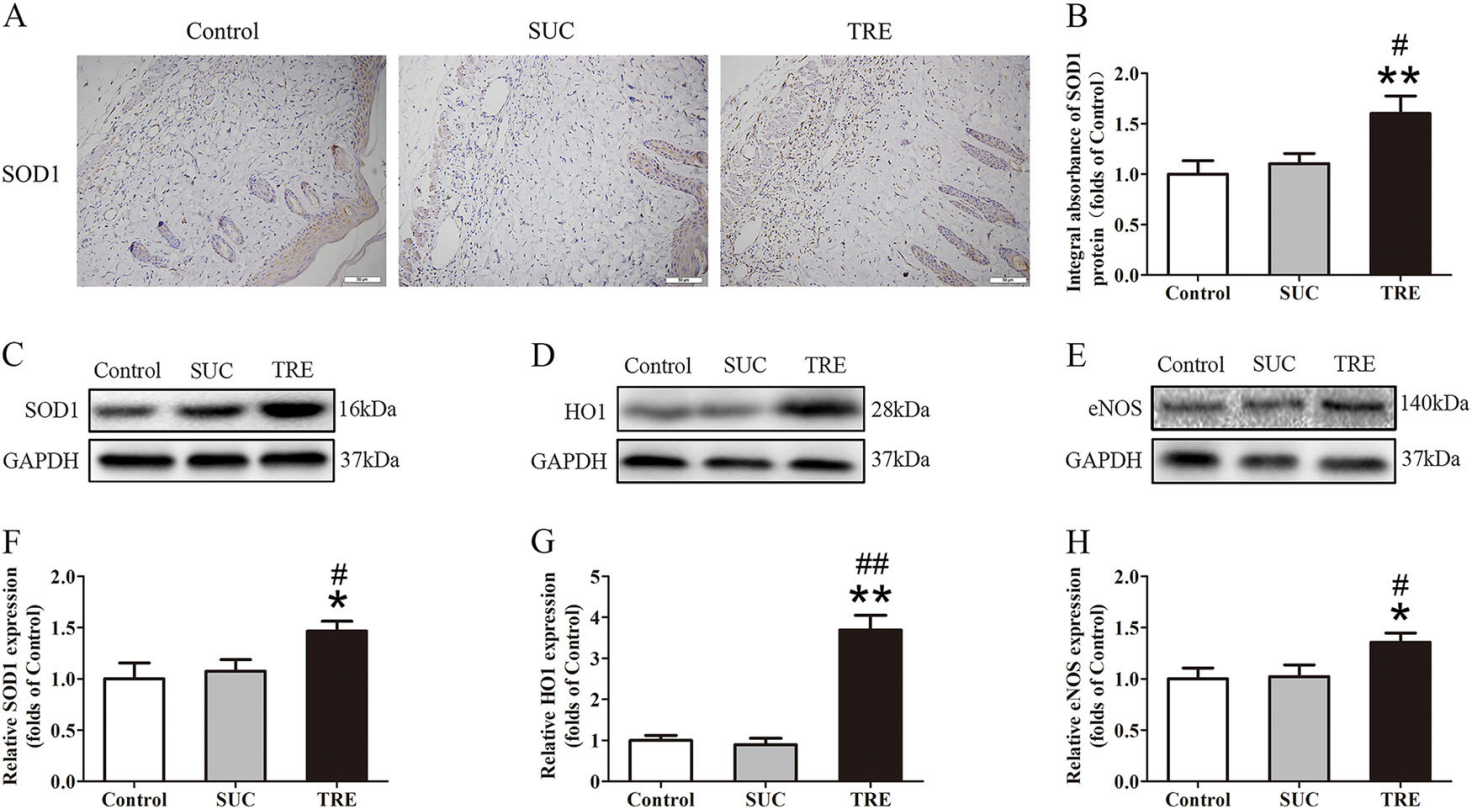
TRE inhibits oxidative stress in random skin flaps. **a** IHC for SOD1 expression in the ischemic flaps of the Control, SUC and TRE groups (original magnification ×200; scale bar, 50 μm). **b** The optical density values of SOD1 were quantified and analyzed in each group. **c**–**e** Western blotting for SOD1, HO1and eNOS expressions in the ischemic flaps of the Control, SUC and TRE groups. The gels have been run under the same experimental conditions, and cropped blots are used here. **f**–**h** Optical density values of SOD1, HO1, and eNOS were quantified and analyzed in each group. Values are exhibited as means ± SEM, *n* = 6 per group. *p < 0.05 and **p < 0.01, vs. Control group; ^#^*p* < 0.05 and ^##^*p* < 0.01, vs. SUC group

Given that oxidative stress plays a major role in flap viability, we also investigated whether trehalose modulates oxidative stress. SOD1 levels were analyzed by IHC and Western blotting of the dermis, and results show that SOD1 levels were higher in the TRE group compared to Control and SUC groups (Fig. 4a, b, c, f). HO-1 and eNOS protein levels were also increased in the TRE group when compared with Control and SUC groups (Fig. 4d, e, g, h). However, there was no significant difference between the Control and SUC groups in the above tests. These data provide evidence that TRE may promote survivability of skin flaps via reducing of oxidative stress.

### TRE activates autophagy in random skin flaps

**Fig. 5:**
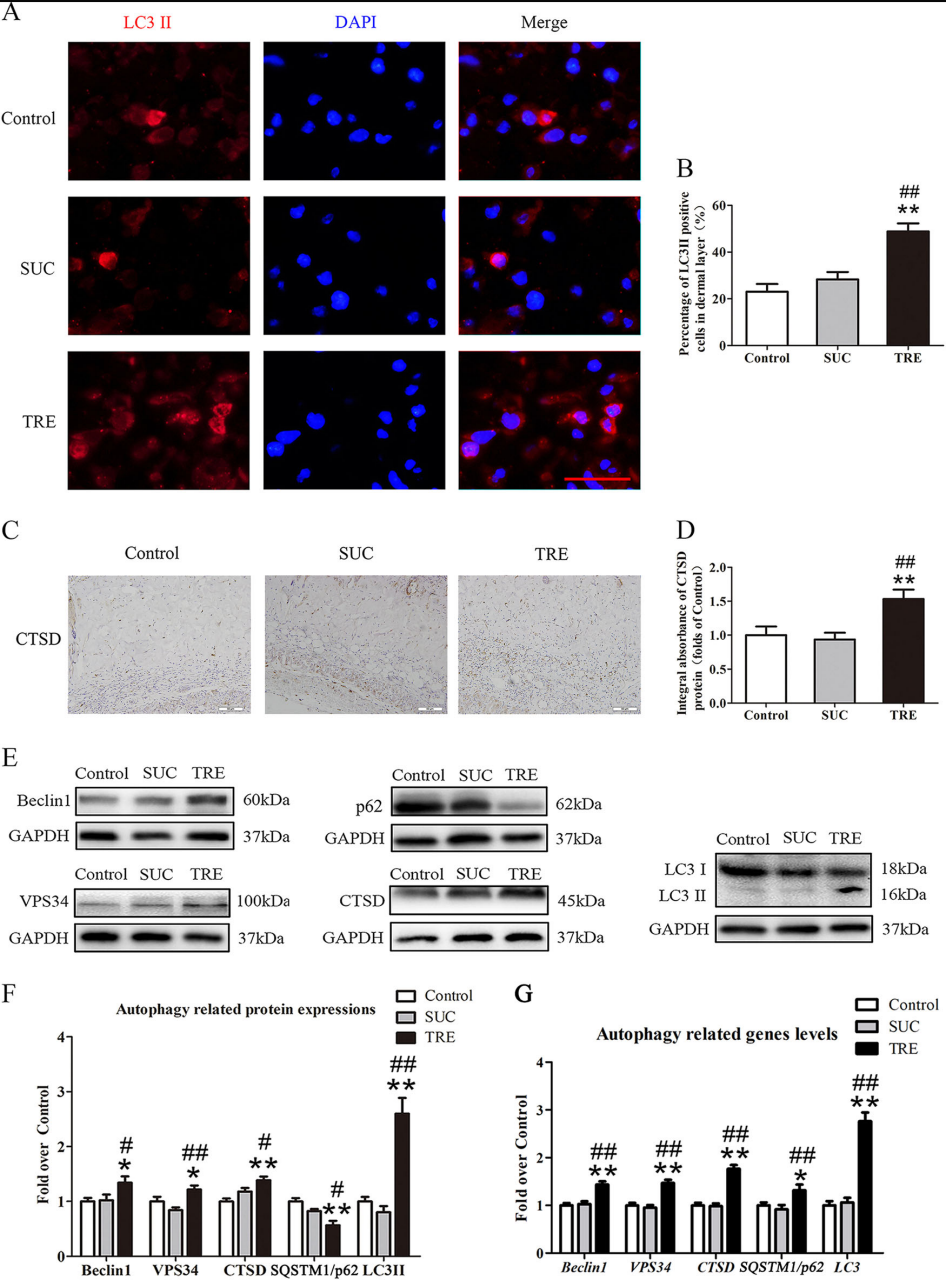
TRE activates autophagy in random skin flaps. **a** Autophagosomes (red) in cells in Area II of flaps in the Control, SUC and TRE groups by Immunofluorescence staining for LC3II (scale bar, 20 μm). **b** The percentages of LC3II positive cells in dermal layer were quantified and analyzed in each group. **c** IHC for CTSD expression in the ischemic flaps of the Control, SUC and TRE groups (original magnification ×200; scale bar, 50 μm). **d** The optical density values of CTSD were quantified and analyzed in each group. **e** Western blotting for Beclin1, VPS34, CTSD, SQSTM1/p62, and LC3II expressions in the ischemic flaps of the Control, SUC and TRE groups. The gels have been run under the same experimental conditions, and cropped blots are used here. **f** Optical density values of Beclin1, VPS34, CTSD, SQSTM1/p62, and LC3II were quantified and analyzed in each group. **g** Transcripts of Beclin1, VPS34, CTSD, SQSTM1/p62, and LC3 genes detected by real-time PCR. Values are expressed as means ± SEM, *n* = 6 per group. *p < 0.05 and **p < 0.01, vs. Control group; ^#^*p* < 0.05 and ^##^*p* < 0.01, vs. SUC group

To evaluate autophagy activation, we quantified Beclin1, VPS34, LC3II, CTSD, and SQSTM1/p62 protein levels. These proteins are direct players in the autophagy process, with Beclin1, VPS34, and LC3II being autophagosomal proteins, CTSD being an autolysosome-related protein, and SQSTM1/p62 being an autophagic substrate protein. The frequency of LC3II-positive cells in the dermis of the TRE group was higher than that in the Control and SUC groups (Fig. 5a, b). IHC analysis revealed higher CTSD levels in the TRE group (Fig. 5c, d), which was also demonstrated by western blotting (Fig. 5e, f). Autophagosomal proteins Beclin 1, VPS34, and LC3II were all upregulated in the TRE group as well (Fig. 5e, f). Finally, autophagic substrate protein SQSTM1/p62 was decreased in response to TRE treatment (Fig. 5e, f). Real-time PCR analysis also demonstrated that the mRNA levels of Beclin 1, VPS34, CTSD, SQSTM1/p62 and LC3 were significantly up-regulated in the TRE group (Fig. 5g). There was no significant difference between the Control and SUC groups in the above tests. These data provide evidence that trehalose is indeed activating autophagy in skin flaps, which may account for the observed inhibition of oxidative stress, decreased apoptosis, and increased angiogenesis.

### Inhibition of autophagy reverses the effects of TRE on angiogenesis, apoptosis, oxidative stress and depresses random skin flap survival

**Fig. 6:**
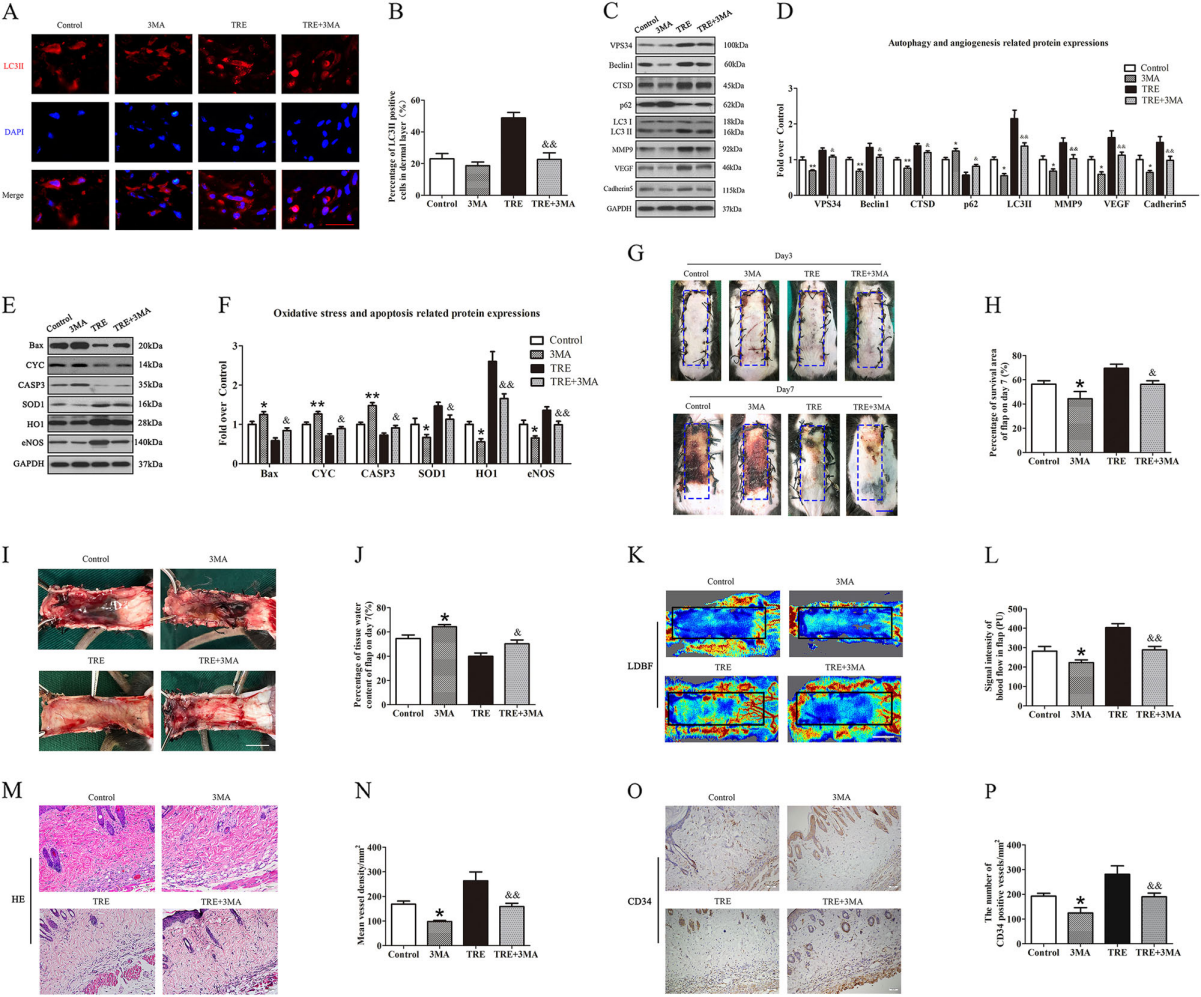
Inhibition of autophagy reverses the effects of TRE on angiogenesis, apoptosis, oxidative stress and flap survival. **a** Autophagosomes(red) in cells in Area II of flaps in the Control, 3MA, TRE and TRE+3MA groups by Immunofluorescence staining for LC3II (scale bar, 20 μm). **b** The percentages of LC3II positive cells in dermal layer were quantified and analyzed in each group. **c**, **e** The expressions of autophagy-related protein VPS34, Beclin1, CTSD, SQSTM1/p62, and LC3II; angiogenesis-related protein MMP9, VEGF, and Cadherin 5; apoptosis-related protein Bax, CYC, and CASP3 and oxidative stress-related protein SOD1, HO1, and eNOS in each group, were assessed by Western blotting. The gels have been run under the same experimental conditions, and cropped blots are used here. **d**, **f** Optical density values of VPS34, Beclin1, CTSD, SQSTM1/p62, LC3II, MMP9, VEGF, Cadherin 5, Bax, CYC, CASP3, SOD1, HO1, and eNOS expressions in each group. **g** Digital photographs of flaps of the Control, 3MA, TRE and TRE+3MA groups on POD 3 and POD 7 (scale bar, 1 cm). **h** The percentages of survival area in each group were quantified and analyzed. **i** Digital photographs of the inner side of flaps in the Control, 3MA, TRE and TRE+3MA groups on POD7 (scale bar, 1 cm). **j** Histogram of percentage of tissue water content in each group. **k** Full field LDBF images of flaps in each group on POD 7 (scale bar, 1 cm). **l** The signal intensity of blood flow of flaps was quantified and analyzed in each group. (**m**) H&E staining to show vessels in area II of flaps in the Control, 3MA, TRE and TRE+3MA groups (original magnifcation ×200; scale bar, 50 μm). **n** Histogram of percentage of MVDs in each group. **o** IHC for CD34 to present vessels of area II in the Control, 3MA, TRE and TRE+3MA groups (original magnifcation ×200; scale bar, 50 μm). **p** Histogram of percentage of CD34-positive vessels in each group. Values are expressed as means ± SEM, *n* = 6 per group. *p < 0.05 and **p < 0.01, vs. Control group; ^&^*p* < 0.05 and ^&&^*p* < 0.01, vs. TRE group

To demonstrate that activation of autophagy is the primary mechanism by which TRE exert its therapeutic effects outlined above, we administered 3MA (an autophagy inhibitor) alone and co-administered 3MA with TRE, then evaluated outcomes. Our results showed that the frequency of LC3II-positive cells in the dermis of animals treated with TRE and 3MA is decreased compared with TRE alone; meanwhile, the mean frequency value in the 3MA group was less than the Control group, although without significance (Fig. 6a, b). Western blotting further revealed increases in SQSTM1/p62, and decreases in VPS34, Beclin 1, CTSD, and LC3 II levels in the TRE+3MA group (Fig. 6c, d) compared with the TRE group. The treatment of 3MA also up-regulated the level of SQSTM1/p62, and down-regulated the expressions of VPS34, Beclin1, CTSD, and LC3II (Fig. 6c, d). Together, these results demonstrate that 3MA inhibits the autophagy activating effects of trehalose in our model. Our results also revealed that expressions of MMP9, VEGF, and Cadherin 5 were significantly downregulated in TRE+3MA mice compared with TRE alone, and downregulated in 3MA mice compared with Control group (Fig. 6c, d). This was also seen for eNOS, SOD1, and HO-1 in TRE+3MA mice compared with TRE alone, and decreased in 3MA mice compared to Control mice (Fig. 6e, f). Conversely, Bax, CYC, and CASP3 were all significantly upregulated in the TRE+3MA group compared with TRE alone, and also upregulated in the 3MA group compared with Control group (Fig. 6c–f). These results indicate that modulation of protein levels by trehalose is significantly impacted when 3MA is co-administered.

Next, we asked whether co-administration of 3MA ablates the therapeutic benefit of trehalose in terms of flap survivability, edema, and angiogenesis. Among the Control, 3MA, TRE and TRE + 3MA groups, flap survival was not significantly different on day 3, but 3MA group showed lower flap survival compared to the Control group on day 7, and TRE+3MA showed significantly lower flap survival than TRE alone on day 7, with clearly increased edema (Fig. 6g–i). 3MA showed higher tissue water content than Control group and TRE+3MA yielded higher tissue water content than TRE alone (Fig. 6j). As shown by LDBF, 3MA alone compromised blood flow compared to the Control group, and TRE+3MA also compromised blood flow compared to TRE alone (Fig. 6k, l). H&E staining showed a decrease in the number of microvessels in the 3MA and TRE+3MA group (Fig. 6m), with significantly lower mean vessel density than the Control and TRE group (Fig. 6n) and fewer CD34-positive vessels (Fig. 6o, p). Together, our investigation suggests that 3MA ablates the autophagy activating effects of TRE, impacts TRE’s modulation of key proteins, and also compromises TRE’s therapeutic effect in the context of skin flaps.

### TRE activates autophagy via enhancing TFEB activity

**Fig. 7:**
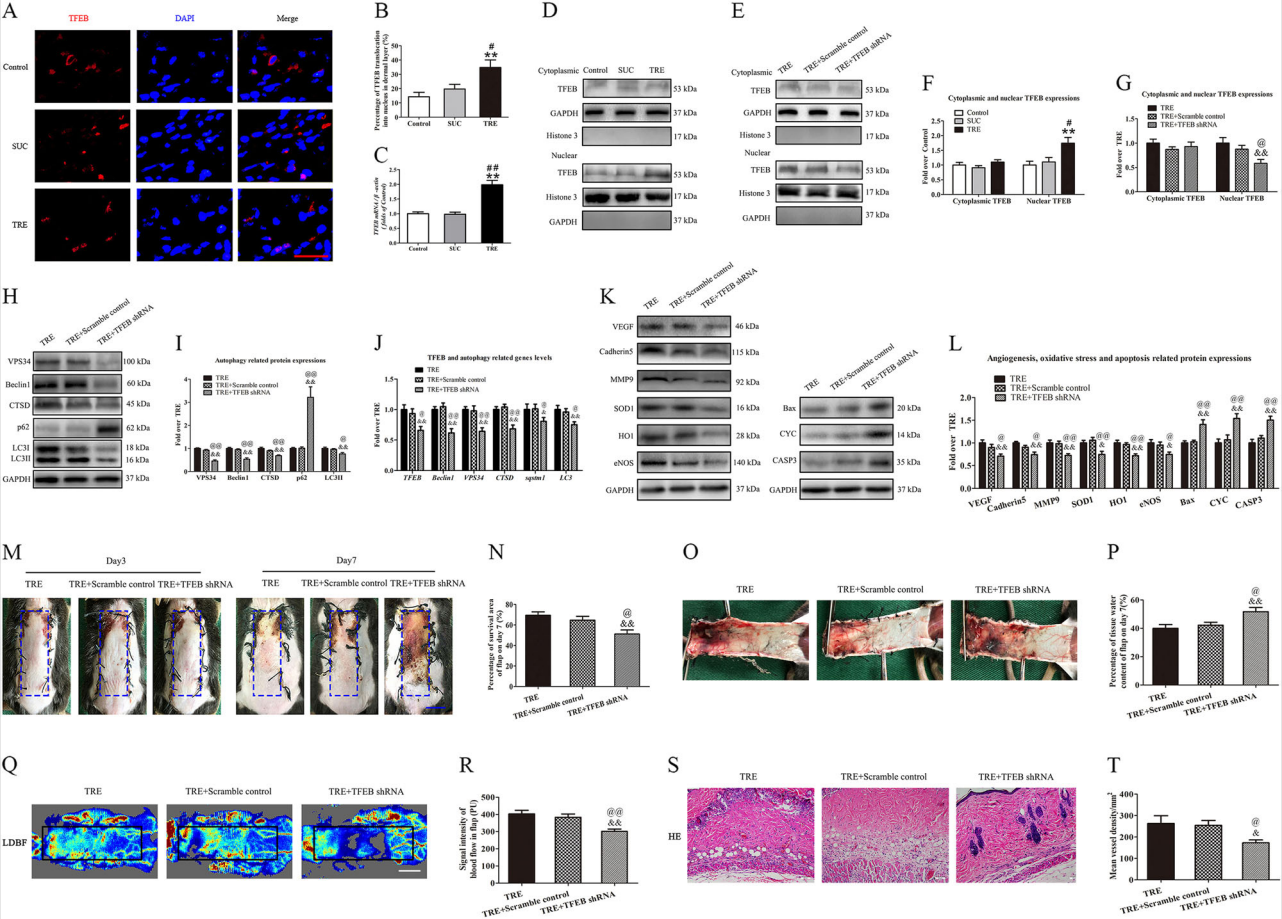
TRE activates autophagy via enhancing TFEB activity. **a** Nuclear translocation of TFEB (red) in cells of flaps in the Control, SUC and TRE groups by Immunofluorescence staining for TFEB (scale bar, 20 μm). **b** The percentages of TFEB translocation into nucleus in dermal layer were quantified and analyzed. **c** Transcripts of TFEB gene detected by real-time PCR. **d** and **e** The expressions of cytoplasmic TFEB, nuclear TFEB in the Control, SUC, TRE, TRE+Scramble control and TRE + TFEB shRNA groups as detected by Western blotting. The gels have been run under the same experimental conditions, and cropped blots are used here. **f**, **g** Optical density values of cytoplasmic TFEB and nuclear TFEB expressions in each group. **h**, **i** The expressions of VPS34, Beclin1, CTSD, SQSTM1/p62 and LC3II in the TRE, TRE+Scramble control and TRE+TFEB shRNA groups as assessed by Western blotting. And optical density values of these autophagy-related protein expressions in each group. **j** Transcripts of TFEB and its targeting genes, including Beclin1, VPS34, CTSD, SQSTM1/p62, and LC3 genes, were detected by real-time PCR. **k**, **l** The expressions of angiogenesis-related protein VEGF, Cadherin 5 and MMP9; oxidative stress-related protein SOD1, HO1, and eNOS and apoptosis-related protein Bax, CYC, and CASP3 in each group, were assessed by Western blotting. And optical density values of these protein expressions in each group. **m** Digital photographs of flaps of the TRE, TRE+Scramble control and TRE+TFEB shRNA groups on POD 3 and POD 7 (scale bar, 1 cm). **n** The percentages of survival area in the TRE, TRE+Scramble control and TRE+TFEB shRNA groups were quantified and analyzed. **o** Digital photographs of the inner side of flaps in the TRE, TRE+Scramble control and TRE+TFEB shRNA groups on POD7 (scale bar, 1 cm). **p** Histogram of percentage of tissue water content in each group. **q** Full field LDBF images of flaps in each group on POD 7 (scale bar, 1 cm). **r** The signal intensity of blood flow of flaps was quantified and analyzed in each group. **s** H&E staining to show vessels in area II of flaps in the TRE, TRE + Scramble control and TRE+TFEB shRNA groups (original magnifcation ×200; scale bar, 50 μm). **t** Histogram of percentage of MVDs in each group. Values are expressed as means ± SEM, *n* = 6 per group. *p < 0.05 and **p < 0.01, vs. Control group; ^#^*p* < 0.05 and ^##^*p* < 0.01, vs. SUC group; ^&^*p* < 0.05 and ^&&^*p* < 0.01, vs. TRE group; ^@^*p* < 0.05 and ^@@^*p* < 0.01, vs. TRE + Scramble control group

To investigate the mechanism by which trehalose modulates autophagy, we investigated whether TFEB, a known autophagy activator, plays a role. As shown in Fig. 7a, b, the percentage of TFEB translocation into nucleus in dermal layer was increased in the TRE group compared with Control and SUC groups. Real-time PCR analysis showed that the level of TFEB mRNA was significantly upregulated (Fig. 7c), and Western blotting also revealed that the level of nuclear TFEB was upregulated after trehalose treatment (Fig. 7d, f). To explore the role of TFEB in TRE-associated autophagy, the TRE-treated flaps were treated with TFEB shRNA AAV vector, and then levels of nuclear TFEB, autophagy, and flap vitality were assessed. AAV- TFEB shRNA injection significantly decreased TFEB nuclear translocation relative to the TRE and TRE + Scramble control group (Fig. 7e, g), and there was no significant difference between the TRE and TRE + Scramble control groups (Fig. 7e, g). These results suggest that TFEB may play a central role in trehalose’s mechanism of action.

We then evaluated whether TFEB’s nuclear translocation caused by trehalose is responsible for modulation of the autophagy related proteins. Our results demonstrated that VPS34, Beclin1, CTSD, and LC3II were lower in the TRE+TFEB shRNA group when compared to the TRE and TRE+Scramble control groups, with a higher level of p62 (Fig. 7h, i). The expressions of these proteins were not significantly different between the TRE and TRE + Scramble control groups (Fig. 7h, i). Moreover, TFEB and its target gene were detected by real-time PCR. As shown in Fig. 7j, the levels of TFEB, Beclin1, VPS34, CTSD, SQSTM1/p62 and LC3 were significantly down-regulated in the TRE + TFEB shRNA group compared with the TRE and TRE + Scramble control groups. At the same time, our results demonstrated that angiogenesis-related protein VEGF, Cadherin 5 and MMP9 and oxidative stress-related protein SOD1, HO1, and eNOS were lower in the TRE + TFEB shRNA (Fig. 7k, l). Conversely, apoptosis-related protein Bax, CYC, and CASP3 were upregulated in the TRE + TFEB shRNA groups (Fig. 7k, l). The expressions of these proteins and genes were not significantly different between the TRE and TRE + Scramble control group (Fig. 7k, l). Together, these results suggest that nuclear translocation of TFEB due to trehalose administration is responsible for modulation of VPS34, Beclin 1, CTSD, LC3II, and SQSTM1/p62.

Finally, we evaluated whether nuclear translocation of TFEB is responsible for trehalose’s therapeutic benefit in the context of random pattern skin flaps. As shown in Fig. 7m, n, there was no significant difference in the flap survival area among the TRE, TRE+Scramble control, and TRE + TFEB shRNA groups three days after surgery, however, flap survival area was decreased in TRE + TFEB shRNA group relative to the TRE and TRE + Scramble control groups seven days after surgery. Qualitatively, significant edema and subcutaneous venous congestion were appreciated in TRE+TFEB shRNA group, but to a lesser extent in the TRE and TRE+Scramble control groups (Fig. 7o). Quantitatively, the TRE+TFEB shRNA group showed greater water content than the TRE and TRE+Scramble control groups (Fig. 7p). Blood flow in the TRE+TFEB shRNA group was decreased (Fig. 7q), and quantitative blood flow measurements were significantly lower in the TRE+TFEB shRNA group (Fig. 7r). For angiogenesis, we found significantly fewer microvessels in the TRE+TFEB shRNA group (Fig. 7s), with lower mean vessel density than TRE and TRE+Scramble control groups (Fig. 7t). There was no significant difference of the above tests between TRE and TRE+Scramble control groups. Together, these results suggest that trehalose exerts its therapeutic benefit via upregulating autophagy by promoting the nuclear translocation of TFEB.

### TRE regulates Akt signal protein and the AMPK-SKP2-CARM1 signaling pathway

**Fig. 8:**
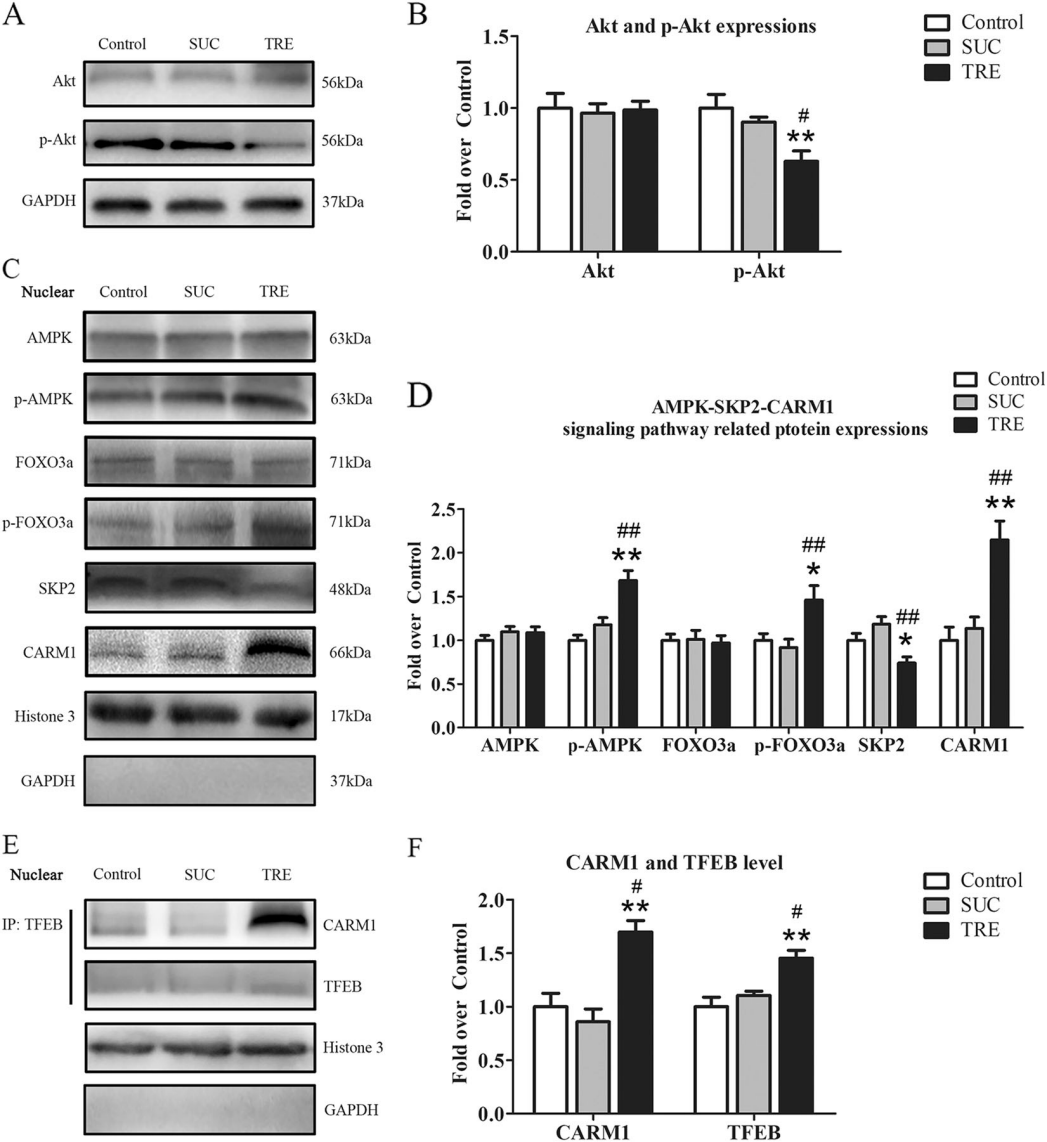
TRE regulates Akt signal protein and the AMPK-SKP2-CARM1 signaling pathway. **a**, **c** The expressions of Akt, p-Akt and AMPK-SKP2-CARM1 signaling pathway-related protein AMPK, p-AMPK, FOXO3a, p-FOXO3a, SKP2 and CARM1 in the Control, SUC and TRE groups as assessed by Western blotting. The gels have been run under the same experimental conditions, and cropped blots are used here. **b**, **d** Optical density values of Akt, p-Akt, AMPK, p-AMPK, FOXO3a, p-FOXO3a, SKP2 and CARM1 expressions in each group. **e** Nuclear CARM1-TFEB complex was detected by co-IP in the Control, SUC and TRE groups. **f** Optical density values of TFEB and CARM1 expressions in each group were quantified and analyzed. Values are expressed as means ± SEM, *n* = 6 per group. *p < 0.05 and **p < 0.01, vs. Control group; ^#^*p* < 0.05 and ^##^*p* < 0.01, vs. SUC group

Finally, we sought to investigate the mechanism by which trehalose signals for TFEB nuclear translocation. Western blotting analysis revealed that the level of phosphorylated Akt in TRE group was decreased relative to the Control and SUC group (Fig. 8a, b). The total level of Akt was not different across these groups, suggesting that Akt signaling is inhibited with trehalose. AMPK, a key responder to starvation and low-energy states, has been shown to be a key regulator in TFEB activity known to be modulated by trehalose. AMPK, phosphorylated FOXO3a, and CARM1 were increased in the TRE group compared with Control and SUC groups (Fig. 8c, d). SKP2 was decreased in the TRE group compared with Control and SUC groups (Fig. 8c, d). Past studies have shown that AMPK activation leads to FoxO3a phosphorylation, which results in reduction of SKP2 and increase of CARM1, increasing CARM1 binding to TFEB promoting TFEB transcription. To further validate that trehalose augments this signaling pathway, we performed co-IP to evaluate the role of TRE in CARM1 and TFEB binding, and our results suggest that TFEB binds to CARM1 at an increased rate in after TRE administration compared with control and SUC groups (Fig. 8e). Furthermore, Co-IP analysis for far western blotting revealed that the levels of TFEB and CARM1 were increased in TRE group compared with Control and SUC groups (Fig. 8f). However, there was no significant difference between the Control and SUC groups in the above tests. Together, these results suggest that trehalose may lead to increased TFEB via reducing Akt signaling and activating the AMPK-SP2-CARM1 pathway.

## Discussions

In this study, we showed that trehalose promotes the survival of random skin flaps via increasing autophagy, leading to angiogenesis of the ischemic areas, inhibition of oxidative stress, and reduction of apoptosis. Mechanistically, we found that TRE induced autophagy by upregulation of TFEB expression via AKT signaling downregulation and AMPK-mTOR-TFEB pathway activation. These findings highlight trehalose’s potential for clinical use to improve outcomes of randam-pattern skin flaps

Random-pattern skin flap is a commonly used tool in reconstructive surgery, and is a form of ischemia flap, of which survival areas is closely related to angiogenesis after flap is established. Previous studies have suggested that promoting angiogenesis prolongs the survival of random skin flaps^9,25^. In our study, treatment with TRE markedly increased microvessels in the dermis of random skin flaps, and flap blood flow was improved. Angiogenesis is a complex process^26^, involving proliferation, migration, and spatial organization of endothelial cells^27^. VEGF is known to increase microvascular permeability and is critical for angiogenesis^28,29^, and, MMP9 is implicated as a major player in VEGF release and the angiogenic switch^30^. Cadherin 5, on the other hand, forms intercellular junctions and prevents disassembly of nascent blood vessels into disorganized endothelial aggregates^31^. Our results demonstrated strong increases of MMP9, VEGF, and Cadherin 5 protein levels after trehalose treatment. Taken together, TRE promotes flap survival due to augmented angiogenesis via increased levels of VEGF, MMP9, and Cadherin 5.

Ischemia is a common complication following skin flap establishment, and with restoration of blood supply, tissue may undergo I/R injury. As a result, excess amounts of reactive oxygen species (ROS) is generated^32^. Trehalose has been reported to respond to high levels of ROS via regulating antioxidant gene expression and the autophagy pathway^33^. In present study, we showed that trehalose increases SOD1, eNOS, and HO1 levels in the flap dermis, suggesting that trehalose inhibits oxidative stress. Apoptosis is an actively regulated form of cell death, which is implicated in numerous physiological and pathological conditions, including I/R injury, and can lead to flap necrosis. TRE has been shown to reduce apoptosis in a various diseases (e.g., chronic ischemic cardiac remodeling^18^, diabetic peripheral neuropathy^34^, and osteoarthritis^35^). Our results showed that trehalose leads to a decrease in apoptosis as measured by apoptosis related proteins Bax, CYC, and CASP3. Together, these results showed that trehalose may improve flap survival due to reducing oxidative stress and inhibiting apoptosis.

Autophagy, the main pathway by which cells clear and degrade intracellular waste, is important for cellular homeostasis, energetics, and host defense^36^. Our previous studies demonstrated that activation of autophagy promotes the survival random skin flaps^8,25,37^. Recently, TRE treatment has been shown to induce autophagy as well as protect against cardiac chronic ischemic remodeling^18^, atherosclerosis^16,20^, and motoneuron degeneration^38^. The process of autophagy is extensively studied, and involves three sequential steps: sequestration, transportation, and degradation. In brief, during sequestration, levels of VPS34, Beclin1, and LC3II are increased^39^^,40^, and during transportation and degradation, stimulation of autophagy flux causes depletion of SQSTM1/p62^41^. CTSD, a lysosomal aspartyl protease, plays an important role during the degradation stage^42^. In the present study, we found that after trehalose treatment, skin flap levels of Beclin 1, VPS34, LC3II, and CTSD were increased and SQSTM1/p62 was decreased, suggesting that trehalose augments autophagy. Furthermore, 3MA, a potent autophagy inhibitor, reversed all the therapeutic benefit of trehalose on flap survival and outcomes, suggesting that autophagy is responsible for trehalose’s beneficial effects on skin flaps.

It is believed that autophagic responses to stress conditions depend on transcriptional regulation^43,44^. TFEB is a known master transcriptional regulator of autophagy^45,46^, and can control autophagosome and lysosome biogenesis and promote autophagosome–lysosome activity by binding to the CLEAR (Coordinated Lysosomal Expression and Regulation) element on a variety of autophagy and lysosomal genes to induce their expression^47^. During stress, inactive TFEB dephosphorylates and translocates to the nucleus to act on its target genes^48^ to promote the transcription of numerous lysosomal and autophagy genes^49,50^. TRE has been shown to enhance activity of autophagy via increasing activation of TFEB^51^. Here, our results showed that TRE treatment significantly increased the percentage of TFEB translocation into nucleus. We also found that AAV-TFEB shRNA, an inhibitor of TFEB translocation, significantly attenuated the effects of TRE on TFEB nuclear translocation and downregulated the level of autophagy. AAV-TFEB shRNA also reversed the effects of TRE on the viability of random skin flaps. These findings indicate that TRE upregulates autophagy and improves flap survival through enhancing the nucleus translocation of TFEB in ischemic flaps.

TFEB activity is regulated at various levels including posttranslational modifications and protein-protein interactions^52^. Promotion of TFEB dephosphorylation can enhance TFEB nucleus translocation and upregulate the TFEB activity. Past studies have shown that inhibition of Akt activity using pharmacological inhibitors promotes nuclear translocation of TFEB and activates the autophagy-lysosome pathway^53^. Once TFEB translocates into nucleus, active TFEB is regulated by AMPK-SKP2-CARM1 signaling pathway^54^. Specifically, AMPK is activated by a decrease in the ATP/ADP ratio under nutrient starvation, leading to FOXO3a phosphorylation and repression of SKP2 transcription^54,55^. Downregulation of SKP2 stabilizes CARM1^56^, which then binds to and activates TFEB^55^. In the present study, we observed that TRE treatment leads to inhibition of Akt activity along with activation the AMPK-SKP2-CARM1 signaling axis, together suggesting that trehalose acts via combinatorial modulation of these signaling pathways. Worthy of note, past research have shown that calcineurin may also be responsible for TFEB de-phosphorylation and nuclear translocation^38,46^. Future studies that explore the role of calcineurin in trehalose-mediated activation of autophagy further elucidate trehalose’s mechanism of action.
